# Structural insights into substrate binding, residue contributions, and catalytic mechanism of phosphopantetheine adenylyltransferase from *Helicobacter pylori*

**DOI:** 10.1042/BSR20241405

**Published:** 2025-08-26

**Authors:** I-Ting Ko, Yi-Ting Yuan, Cheng-Ju Hsieh, Hui-Ting Hsu, Hsien-Sheng Yin

**Affiliations:** 1 Institute of Bioinformatics and Structural Biology, College of Life Sciences and Medicine, National Tsing Hua University, Hsinchu, 30013, Taiwan; 2 Veterinary Research Institute, Ministry of Agriculture, New Taipei City, 25158, Taiwan; 3 Department of Life Science, National Tsing Hua University, Hsinchu, 30013, Taiwan

**Keywords:** crystallography, enzyme kinetics, biophysics

## Abstract

Phosphopantetheine adenylyltransferase (PPAT; EC 2.7.3.3) is a key enzyme in coenzyme A (CoA) biosynthesis. It catalyzes the reversible transfer of an adenylyl group from ATP to 4′-phosphopantetheine (Ppant), producing pyrophosphate and 3′-dephospho-CoA (dPCoA). Although the crystal structures of PPATs with various ligands have been studied, the specific contributions of residues to catalytic efficiency remain unclear. Here, we present the crystal structures of *Helicobacter pylori* PPAT (*Hp*PPAT) in its apo form and complexes with Ppant and ATP. Additionally, we report the structure of the *Hp*PPAT P8A mutant bound to dPCoA, providing the first complete-occupancy structure of a PPAT complex across the hexamer. In the *Hp*PPAT:ATP complex structure, critical active-site residues Thr10, His18, Arg88, and Arg91, conserved in *Escherichia coli* PPAT (*Ec*PPAT), are identified. *Hp*PPAT utilizes Pro8, Lys42, and Arg133 for ATP binding. This differs from the binding pattern observed in other bacterial PPATs. Mutations of these residues, except for Thr10 and Lys42, resulted in a complete loss of enzymatic activity. This result highlights their critical roles. Mutating Thr10 and Lys42 to alanine reduced catalytic efficiency compared with WT *Hp*PPAT but retained substantial activity. These residues are expected to orient the nucleophile for an in-line displacement mechanism. Based on structural studies and mutagenesis data with kinetic measurements and insights from other bacterial PPATs, we propose a refined catalytic mechanism for *Hp*PPAT that emphasizes species-specific active-site interactions. This mechanism provides a foundation structure-based drugs against *H. pylori* infections.

## Introduction

**Figure 1:**
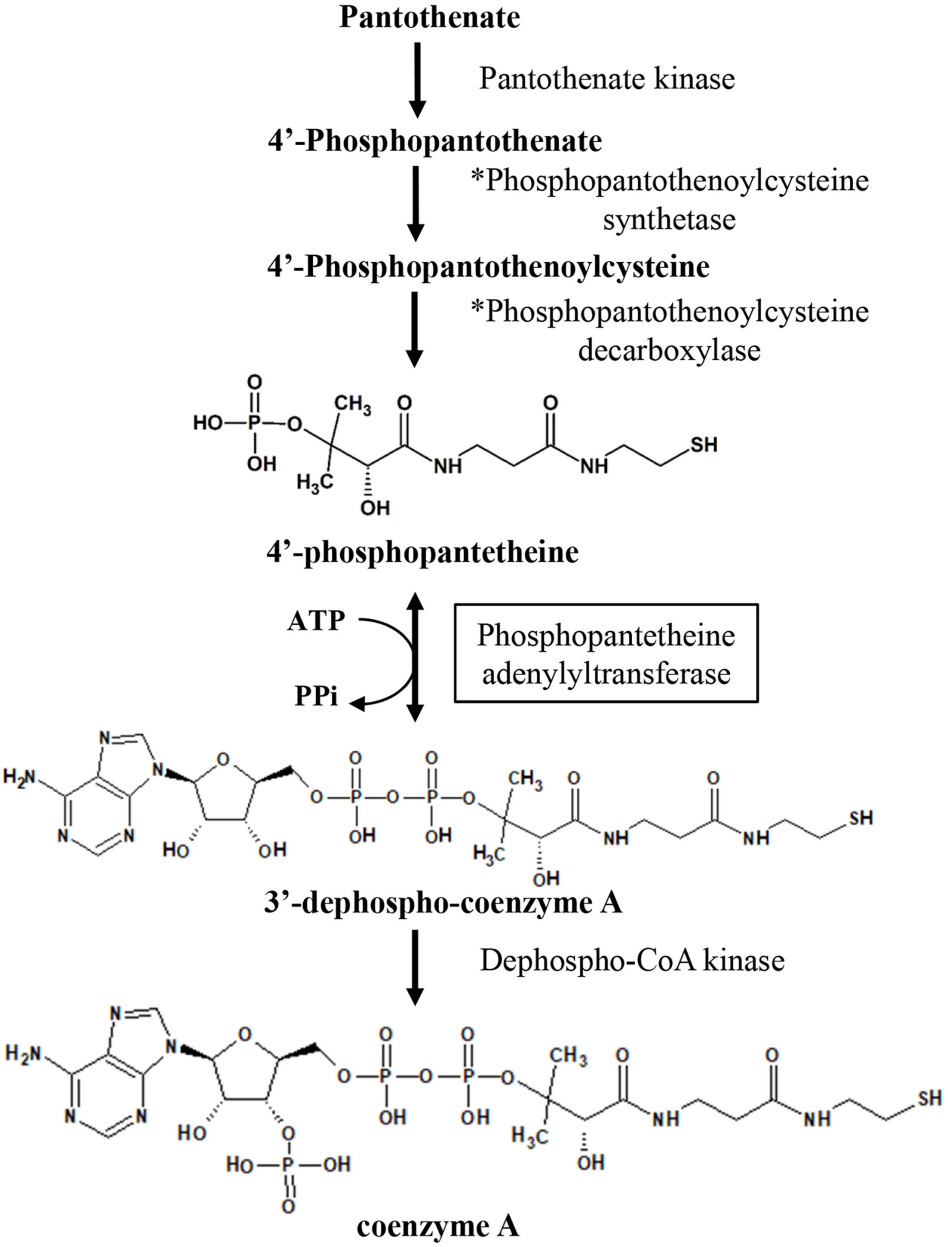
The biosynthetic pathway of coenzyme A in bacteria is illustrated, emphasizing the step catalyzed by phosphopantetheine adenylyltransferase (PPAT), highlighted in a black box. The asterisk (*) indicates that bacteria utilize a single-gene product to catalyze reactions during these two steps.

*Helicobacter pylori* (*H. pylori*) is implicated in various gastrointestinal disorders, including chronic gastritis, peptic ulcer disease, stomach cancer, and mucosa-associated lymphoid tissue lymphoma [1,2]. Treatment typically involves proton pump inhibitors and antibiotics [3], but antibiotic resistance presents significant challenges [4]. Coenzyme A (CoA) is an essential metabolic cofactor in all living organisms, crucial for diverse metabolic pathways, including biosynthesis, degradation, and energy production, such as citric acid cycle and fatty acid synthesis [5]. Bacterial enzyme phosphopantetheine adenylyltransferase (PPAT; EC 2.7.3.3) catalyzes the reversible transfer of an adenylyl groups from ATP to 4′-phosphopantetheine (Ppant), yielding 3′-dephospho-CoA (dPCoA) and pyrophosphate in the penultimate step of CoA biosynthesis (Figure 1) [6]. An inhibitor specific to *Escherichia coli* PPAT (*Ec*PPAT) has been developed [7], which does not inhibit porcine PPAT. Additionally, the PPAT domain of the human bifunctional PPAT/dephospho-CoA kinase protein shows very low sequence similarity to any bacterial PPATs and may differ significantly in overall structure [8]. Genetic footprinting studies have demonstrated that inhibiting *Ec*PPAT prevents *E. coli* growth [9], supporting the idea that bacterial PPAT may be a target for developing new antibacterial drugs.

Crystal structures of bacterial PPATs have been determined in their apo forms [10,11] and in complexes with substrates such as ATP, Ppant [11–14], dPCoA [15], and CoA [16,17]. These structures reveal a core fold featuring five parallel β-strands and four α-helices. The β-pleated-sheet configuration forms a dinucleotide-binding motif fundamental to the enzyme’s ability to bind these substrates, with CoA as a negative-feedback regulator. To delve deeper into the catalytic mechanism of PPAT, researchers examined the crystal structures of *Ec*PPAT in complex with ATP and Ppant, which are consistent with the 4′-phosphate of Ppant undergoing a nucleophilic attack on the α-phosphate of ATP in an in-line displacement mechanism. The enzyme probably stabilizes the pentacovalent transition state by orienting the nucleotide, with His18 forming a crucial hydrogen bond. Lys42 and Thr10 may help orient the nucleophile, while Arg91, Ser128, Ser130, and the amide group of Ser129 may stabilize the β- and γ-phosphate groups of ATP [12].

Despite these advances, our previous research indicates distinct differences in the crystallographic data on the interactions between *H. pylori* PPAT (*Hp*PPAT) and CoA compared with those observed in *Ec*PPAT. In *Hp*PPAT, the residues Thr10, Lys42, Arg88, and Tyr98 form hydrogen bonds with the CoA phosphate group, while *Ec*PPAT uses Asp95 and Glu99 for similar interactions. Additionally, in *Hp*PPAT, Thr15, Gly17, Ile127, and Arg133 interact with CoA, changing its adenine ring orientation [18]. These findings suggest distinct characteristics of the amino acids involved in substrate binding, such as ATP and Ppant, and the catalytic processes of *Hp*PPAT. Although efforts have been made to characterize PPAT kinetics in *E. coli* and *Mycobacterium tuberculosis* [13,19], the impact of specific active-site residues on catalytic efficiency remains unresolved. Furthermore, our experimental results demonstrate that the *Hp*PPAT P8A mutant exhibits higher ATP production in the reverse reaction than the WT *Hp*PPAT, suggesting enhanced apparent activity in dPCoA utilization. Detailed biochemical analyses supporting this observation are presented in the supplementary file.

Based on these findings, the present study aims to clarify the structural basis for substrate-binding residues contribution to *Hp*PPAT’s catalytic efficiency and to unravel its enzymatic mechanism through detailed structural analysis and mutagenesis. X-ray crystallography was used to determine the crystal structures of *Hp*PPAT in its apo form, the ATP- and Ppant-bound complexes of *Hp*PPAT, and the dPCoA-bound complex of the P8A mutant. Site-directed mutagenesis was used to introduce mutations to key active-residues and assess the impact of these mutants on catalytic efficiency. These structures provide important insights into *Hp*PPAT’s catalytic center. Combined structural and mutagenesis analyses facilitate a more in-depth understanding of the *Hp*PPAT enzyme reaction mechanism.

## Results and discussion

### Overall structures

We determined the crystal structures of HpPPAT and its mutant P8A in four distinct forms. The apo structure was resolved at 1.98 Å, and the complexes with ATP, Ppant, and dPCoA were obtained at 2.12 Å, 1.96 Å, and 2.00 Å, respectively (Table 1). Data collection and refinement statistics, including stereochemical quality assessed using SFCHECK [20] and Ramachandran plot analysis [21], are summarized in Table 1. Both the apo *Hp*PPAT and its Ppant-bound form are dimeric. The oligomerization states of *Hp*PPAT and other bacterial PPATs reveal significant structural variations. *Hp*PPAT:ATP assembles as a dodecamer, whereas *Ec*PPAT:ATP forms a dimer [12], and *Enterococcus faecalis* PPAT (*Ef*PPAT:ATP) exists as a hexamer in the ATP-bound state [11]. In the Ppant-bound state, *Hp*PPAT:Ppant and *Ec*PPAT:Ppant adopt a dimeric form, while *Mycobacterium tuberculosis* PPAT (*Mt*PPAT):Ppant assembles as a tetramer [13]. In *Hp*PPAT P8A:dPCoA is a hexamer, whereas *Ec*PPAT:dPCoA remains a dimer. Like *Ec*PPAT, the substrate Ppant binds to only one monomer within the *Hp*PPAT dimer. In the ATP-bound form, *Hp*PPAT assembles as a dodecamer, with complete electron density for ATP observed in chains I, J, and L. In contrast, chains K, G, and H exhibit fragmented electron density, indicating partial ATP occupancy rather than degradation, as no additional density corresponding to hydrolysis products (e.g., ADP or AMP) was detected. Representative electron density maps for the fully occupied L chain and the partially occupied K chain are shown in Supplementary Figure S1. The second hexamer lacks apparent electron density for ATP, which may suggest the difference in local structural variations within the dodecameric assembly.

**Table 1:** Data collection and refinement statistics

Dataset	PPAT apo	PPAT:ATP	PPAT:Ppant	P8A:dPCoA
Synchrotron beamline	NSRRC 13B	NSRRC 13B	NSRRC 13B	NSRRC 13B
Wavelength (Å)	1.0000	1.0000	1.0000	1.0000
Space group	*H*3	*P*1	*H*3	*P*2_1_2_1_2
Unit cell dimensions				
*a, b, c* (Å)	78.74, 78.74, 147.17	67.15, 82.06, 105.51	79.07, 79.07, 147.27	95.89, 126.90, 89.98
α, β, γ (°)	90.00, 90.00, 120.00	88.02, 73.55, 87.14	90.00, 90.00, 120.00	90.00, 90.00, 90.00
Resolution (Å)	30–1.98 (2.03–1.98)	50–2.12 (2.20–2.12)	30–1.96 (2.03–1.96)	30–2.0 (2.05–2.00)
No. of unique reflections	23,881	108,761	24,685	75,207
No. of observations	138,169	463,478	138,169	398,220
Completeness (%)	99.9 (100)	97.8 (94.3)	99.9 (100)	96.3 (97.9)
Redundancy	5.6 (5.6)	2.0 (1.8)	5.6 (5.6)	2.3 (2.2)
Mean I/σ (I)	37.0 (3.9)	18.5 (1.4)	37.0 (3.9)	21.6 (2.4)
Protein mol/AU	2	12	2	6
*R*_merge_	0.045 (0.462)	0.077 (0.920)	0.045 (0.462)	0.047 (0.454)
Refinement				
Resolution (Å)	27.0–1.97 (2.03–1.97)	27.10–2.12 (2.20–2.12)	27.10–1.96 (2.03–1.96)	27.10–2.00 (2.03–2.00)
No. reflections used	21,969 (1,226)	102,141 (5,330)	23,339 (5,958)	68,551 (3,661)
No. atoms				
Protein	2484	1,4854	2484	7350
Ligands	0	93	22	264
Solvent	67	249	96	619
Protein B-factors (Å)	27.1	43.9	20.8	28.3
Ligand B-factors (Å)		78.5	25.0	26.9
Solvent B-factors (Å)	28.0	39.2	22.2	43.8
*R*_work_/*R*_free_ (%)	17.94/23.08	20.51/24.62	15.96/21.92	19.56/25.02
R.m.s deviations				
Bond lengths (Å)	0.011	0.017	0.011	0.02
Bond angles (°)	1.832	1.911	1.775	2.003
Ramachandran plot				
Favored (%)	98.7	97.7	98.7	98.7
Allowed (%)	1.3	2.3	1.3	1.1
Outliers (%)	0	0.1	0	0.2
PDB entry	8XSK	8XRW	8XSC	8XSV

Number in parenthesis corresponds to the highest resolution shell (2.05–2.00) Å.

We performed a C-alpha root mean square deviation (RMSD) analysis comparing fully occupied (chain L) and partially occupied monomers (chains K, G, and H) to investigate whether ATP-binding differences are associated with distinct conformational states. The calculated RMSD values indicate minimal structural deviations between the fully occupied monomer (chain L) and the partially occupied monomers (chain K: 0.679 Å, chain G: 0.746 Å, and chain H: 0.500 Å), suggesting that the observed ATP-binding differences are not due to large-scale conformational changes. Instead, these variations are likely attributable to differences in local ligand stabilization or crystal-packing effects rather than distinct structural states. Electron density maps further support this interpretation by visually demonstrating the weaker ATP density in partially occupied monomers (Supplementary Figure S1).

**Figure 2:**
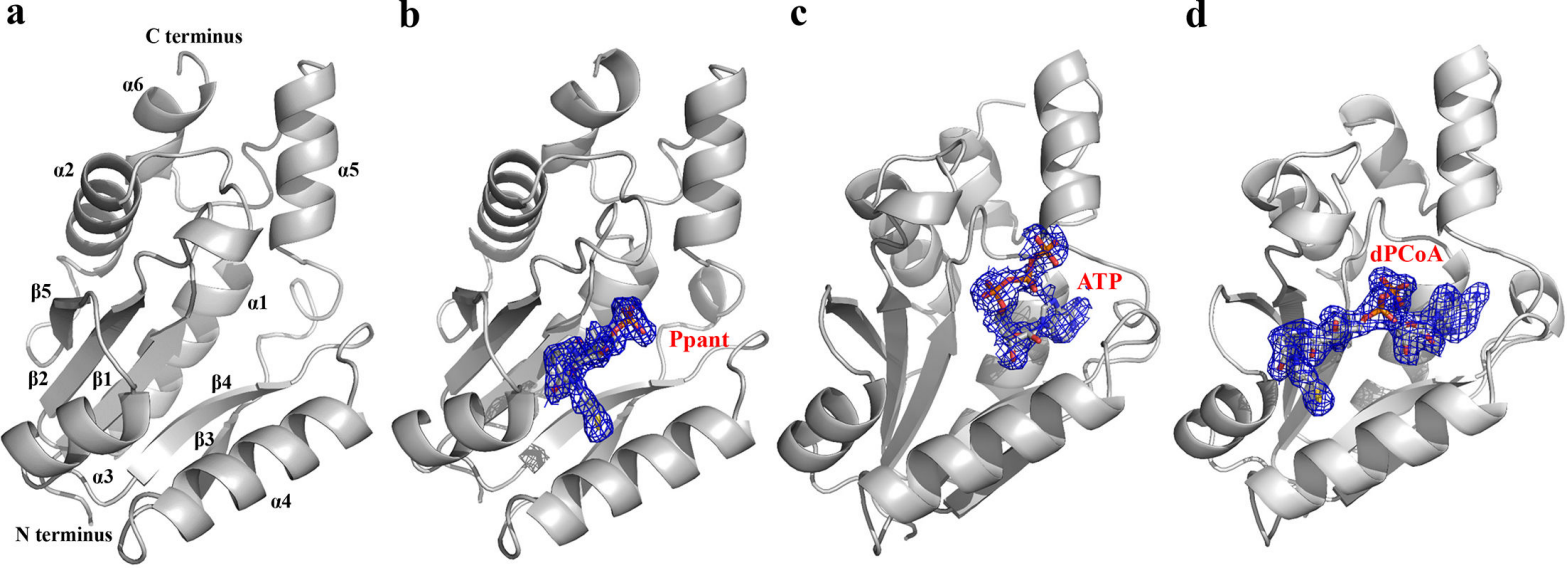
Monomeric structures of *Hp*PPATs in various states. (**a**) Ribbon diagram of the apo *Hp*PPAT monomer. (**b–d**) Ribbon diagrams of *Hp*PPAT monomers bound to ATP, Ppant, and P8A mutant bound to dPCoA. The (*2F_o_-F_c_*) electron density maps clearly show the presence of ATP, Ppant, and dPCoA in their bound forms.

Despite these ATP-occupancy variations, the monomeric architecture of *Hp*PPAT remains consistent across different ligand-bound states. Each apo, ATP-bound, and Ppant-bound monomer comprises 157 residues, featuring a five-strand parallel β-sheet and six α-helices (Figure 2). These structures include 67, 254, and 97 water molecules (Table 1). The overall fold is consistent with that seen in CoA-bound *Hp*PPAT [18], where the parallel β-sheet forms a dinucleotide-binding fold [22], enabling *Hp*PPAT to bind ATP, Ppant, and dPCoA. Distinct electron density maps confirmed the presence of these ligands in the bound structures (Figure 2b–d).

#### Structural comparison of the substrate-binding sites of *H. pylori* and other bacterial PPATs

**Figure 3:**
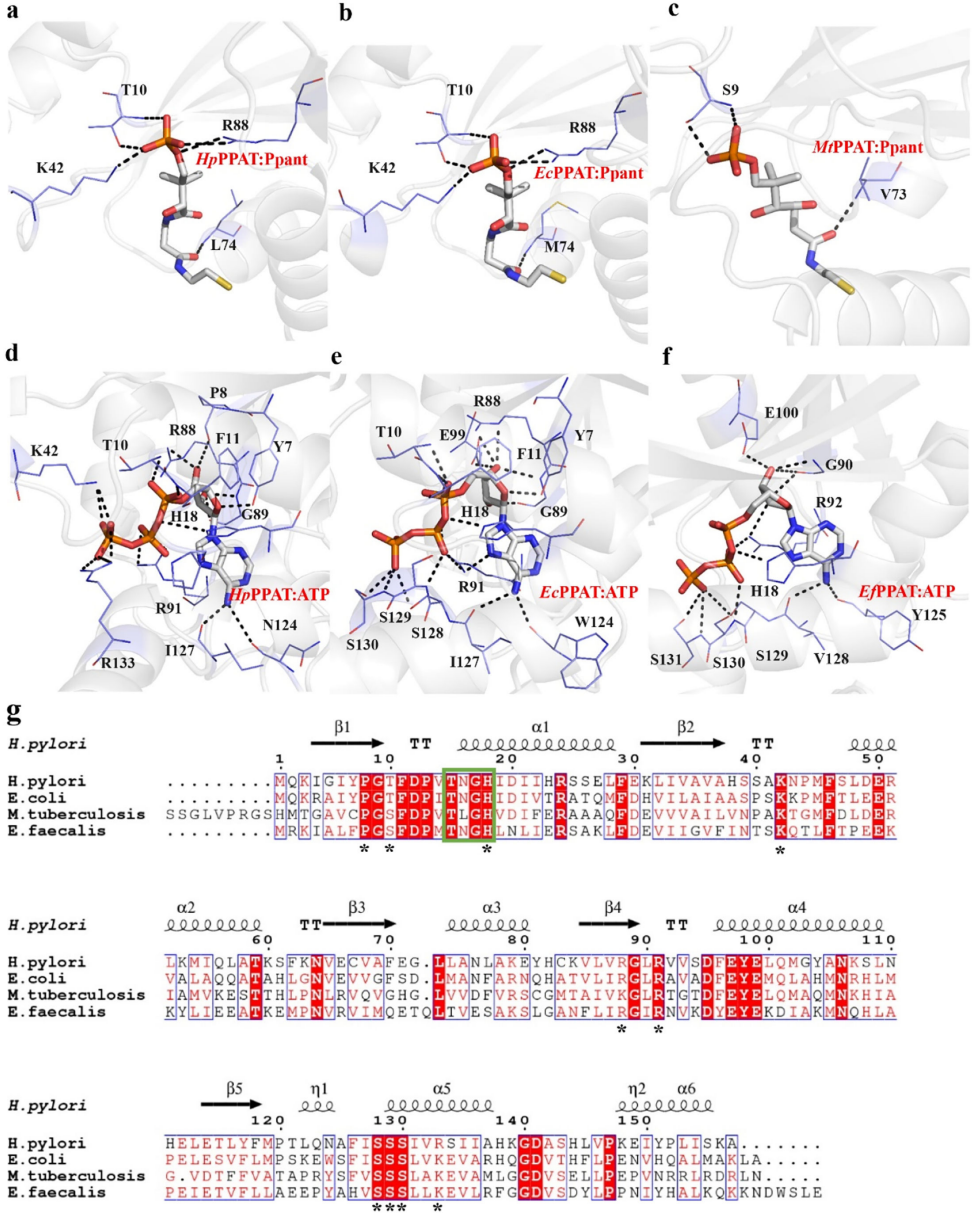
Three-dimensional structure of *Hp*PPATs. (**a–c**) Characterization of the Ppant/PPAT interfaces for *H. pylori* (*Hp*PPAT:Ppant), *E. coli* (*Ec*PPAT:Ppant), and *M. tuberculosis* (*Mt*Ppant), highlighting residues involved in Ppant binding. Ppant stick models are included in each diagram. (**d–f**) Characterization of the ATP/PPAT interfaces in *H. pylori* (*Hp*PPAT:ATP), *E. coli* (*Ec*PPAT:ATP), and *E. faecalis* (*Ef*PPAT:ATP), indicating residues involved in ATP binding. ATP stick models are incorporated into each diagram. The structural comparison does not include *E. faecalis* PPAT (*Ef*PPAT:PPAT) with pantetheine or *M. tuberculosis* PPAT (*Mt*PPAT:PPAT) with adenosine-5′-[(α,β)-methyleno]triphosphate. (**G**) Structure-based sequence alignment of PPATs from *H. pylori* (PDB code 8XSK), *E. coli* (PDB code 1GN8), *M. tuberculosis* (PDB code 1FTU), and *E. faecalis* (PDB code 3ND5), created using the crystal structure of *Hp*PPAT apo form as a template. The secondary structure elements and residue numbering shown above the sequences refer to *H. pylori* PPAT: α, α-helix; η, 3₁₀-helix (denoted as η-helix by ESPript) [23]; β, β-sheet; TT, β-turn. Conserved residues are shown as white letters on a red background, partially conserved residues as red letters, and similar residues as blue boxes. The conserved T/HxGH sequence motif responsible for nucleotide binding is highlighted by the green box. Asterisks denote *Hp*PPAT residues P8, T10, H18, K42, K88, R91, S128, S129, S130, and R133 mutated in this study. The sequence alignment is generated with Clustal Omega [24], and the figure is generated with ESPript [23].

We compared the crystal structures of *Hp*PPAT bound to Ppant or ATP with those of PPATs from other bacterial species bound to Ppant or ATP (Figure 3a–f). In the Ppant-bound structure of *Hp*PPAT, residues Thr10, Lys42, Leu74, and Arg88 form hydrogen bonds with the Ppant substrate (Figure 3a), similar to the pattern observed in the *Ec*PPAT complex (PDB ID:1QJC) [12] (Figure 3b). However, in the *Mt*PPAT structure (PDB ID:3NBK) [13], only Val73 forms a hydrogen bond with Ppant (Figure 3c). Despite sequence alignment showing that *Mt*PPAT Val73 and *Hp*PPAT Leu74 are equivalent (Figure 3g), this difference suggests species-specific adaptations in PPATs, even with common elements in Ppant binding.

For the ATP-bound structure of *Hp*PPAT, residues His18, Gly89, Arg91, Asn124, and Ile127 form hydrogen bonds with ATP, similar to their counterparts in *E. faecalis* (Gly90, Arg92, Tyr124, and Val128) (Figure 3d–f). Additionally, Tyr7, Thr10, Phe11, and Arg88 interact with ATP, consistent with *Ec*PPAT interactions (Figure 3d and e), highlighting conserved ATP-binding mechanisms across bacterial PPATs. A feature specific to the *Hp*PPAT–ATP complex is the involvement of Pro8, Lys42, and Arg133 in hydrogen bonding with ATP, which is not observed in *Ec*PPAT or *Ef*PPAT (Figure 3d and e). In contrast with *Ec*PPAT’s Ser128, Ser129, and Ser130 forming hydrogen bonds with ATP’s γ-phosphate and β-phosphate groups, these residues do not form hydrogen bonds in the *Hp*PPAT–ATP complex (Figure 3d). Figure 3g provides sequence alignment of bacterial PPATs, demonstrating high sequence similarity across species. Notably, catalytic residues are nearly identical, and this study’s residues involved in mutagenesis are notably marked with an asterisk. Despite the overall sequence similarity among bacterial PPATs, the distinct ATP-binding behavior observed in *Hp*PPAT may reflect species-specific adaptations. Further analysis is needed to fully elucidate the precise roles of the catalytic center residues in *Hp*PPAT in order to fully understand its catalytic activity.

To further examine structural conservation and variations among bacterial PPATs, we performed RMSD analysis on C-alpha alignments. The comparison between *Hp*PPAT:ATP and *Ec*PPAT:ATP showed an RMSD of 1.246 Å, indicating high structural similarity. RMSD values were calculated using Cα atoms across all structurally aligned residues. In contrast, alignment with *Ef*PPAT:ATP yielded a higher RMSD of 3.240 Å, suggesting substantial differences in ATP binding. A similar correlation was observed in the Ppant-bound structures, where *Hp*PPAT:Ppant and *Ec*PPAT:Ppant exhibited substantial structural similarity, with an RMSD of 1.056 Å. In comparison, alignment with *Mt*PPAT:Ppant produced a higher RMSD of 4.857 Å. Additionally, comparing *Hp*PPAT P8A:dPCoA with *Ec*PPAT:dPCoA exhibited an RMSD of 1.133 Å.

**Figure 4:**
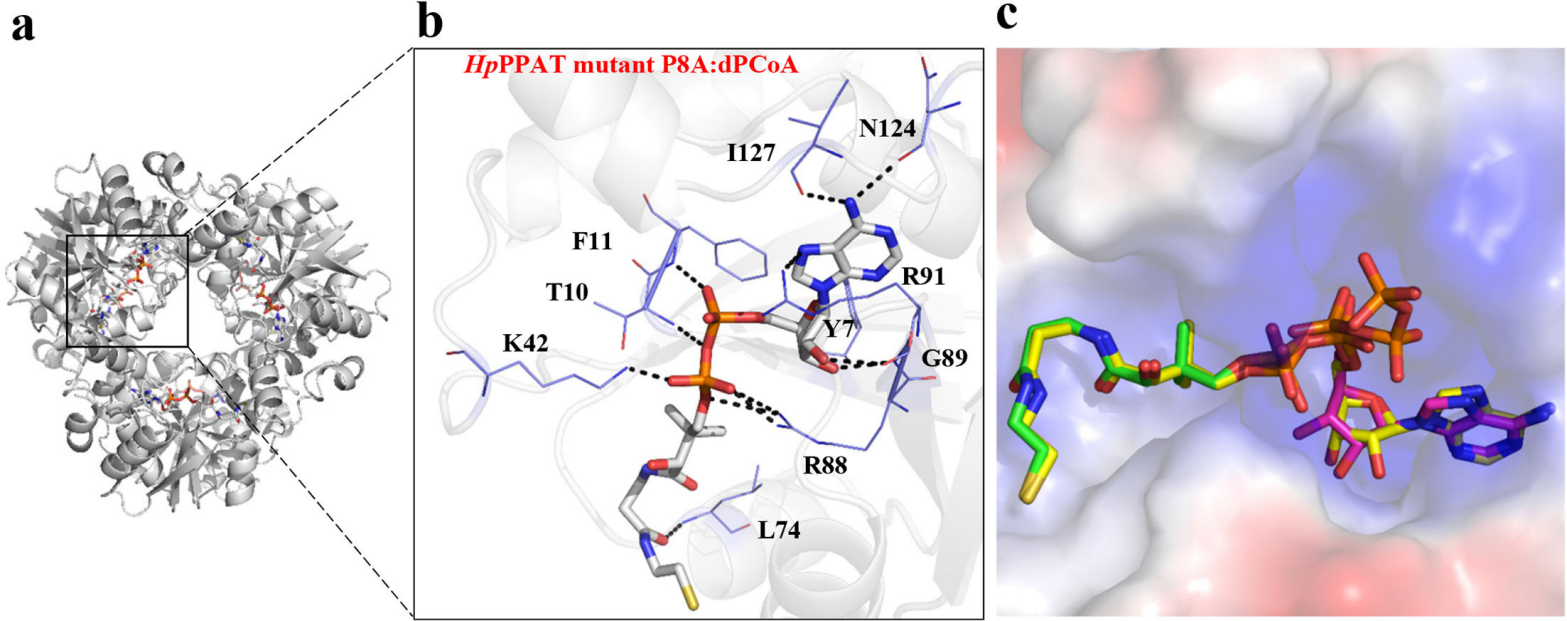
(**a**) Crystal structure of the *H. pylori* PPAT mutant (P8A) hexamer in complex with dPCoA in each monomer (PDB code 8XSV). (**b**) Close-up view of hydrogen-bonding interactions between one monomer of P8A monomer and dPCoA. The interaction map provides detailed information, with dPCoA represented in a stick model and interacting residues depicted in purple lines. Black dotted lines indicate polar hydrogen bond contacts. (**c**) Crystal structure of *Hp*PPAT in a ternary complex with substrates, 4′-phosphopantetheine (Ppant) and ATP, resolved at 1.96 Å and 2.12 Å, respectively (PDB codes 8XSC and 8XRW, respectively). Ppant is shown in green; red stick models represent ATP. Using surface electrostatics, the active site of HpPPAT is displayed as a space-filling model, colored based on surface electrostatics. Structural superposition with *Hp*PPAT mutant P8A:dPCoA (yellow stick model) demonstrates close agreement between substrate and product positions

Additionally, our data indicate that the *Hp*PPAT P8A mutant shows increased ATP production in the reverse enzyme reaction relative to WT *Hp*PPAT under the tested conditions (Supplementary Figure S2), which may suggest enhanced apparent activity with dPCoA. This increased luminescence signal was not anticipated, as the P8A mutant was initially generated to support crystallization of the *H*pPPAT–dPCoA complex. The result should be considered qualitative and preliminary, as no kinetic parameters were directly measured, and the structural analysis did not reveal pronounced differences in the active site. Accordingly, we consider this observation descriptive and do not interpret it as evidence of enhanced catalytic efficiency. We therefore avoid definitive conclusions regarding catalytic efficiency or binding affinity. However, we recognize that catalytic reversibility is governed by thermodynamic constraints, and therefore, this observation does not imply a shift in equilibrium or direct changes in binding affinity. The crystal structure of the *Hp*PPAT P8A mutant in complex with its major product, dPCoA, was determined at pH 7.5, revealing full occupancy within the hexamer (Figure 4a). This finding contrasts with previously determined PPAT:dPCoA structures at pH 5.0, which exhibited half-the-site reactivity [15] . However, ligand-binding studies at pH 8.0 using isothermal titration calorimetry binding assays demonstrated that ATP, Ppant, dPCoA, and CoA occupied all six monomers of the *E. coli* PPAT hexamer [19], indicating that the full occupancy observed at pH 7.4 in *Hp*PPAT:dPCoA might be a pH-dependent phenomenon. The binding model for dPCoA in *Hp*PPAT is strikingly similar to those observed in *Ec*PPAT (PDB code 1B6T) [15], with critical residues forming conserved interactions (Figure 4b). A superposition of the substrate-bound ternary structures of *Hp*PPAT with ATP and Ppant and the product-bound structure with dPCoA shows close agreement in the positions of substrates and products. ATP, Ppant, and the corresponding moiety in dPCoA overlap entirely at the PPAT active site, highlighting precise substrate positioning within the enzyme’s catalytic pocket (Figure 4c).

#### *Hp*PPAT mutants and kinetic assays

The amino acid sequences of PPATs from *H. pylori*, *E. coli*, *M. tuberculosis*, and *E. faecalis* were aligned (Figure 3g), revealing conserved residues essential for catalysis or substrate binding (Ppant or ATP) [12,25]. PPATs belong to the nucleotidyltransferase α/β phosphodiesterase superfamily, characterized by the TxGH motif, which is crucial for nucleotide binding [26]. This motif is notably conserved, especially in the final histidine residue (His18 in *E. coli* and *H. pylori* PPATs) (Figure 3g, green box). His18 is thought to stabilize the transition state and bind to the α-phosphate of the ATP moiety in the *EcP*PAT transition state [12]. In *Hp*PPAT, His18 also binds to the oxygen in ATP, forming hydrogen bonds that connect the α-phosphate group (Figure 2d). Additionally, the amide groups of Thr10 and Phe11 form hydrogen bonds with the oxygen atoms in the α-phosphate group of ATP (Figure 2d), a feature also observed in *Ec*PPAT:ATP complex structure (Figure 3e). Arg91 in *Hp*PPAT:ATP forms a hydrogen bond with the β-phosphate group of ATP, a conserved interaction observed in *Ec*PPAT:ATP. This interaction likely facilitates the adoption of a functionally favorable conformation for the pyrophosphate moiety [12].

**Figure 5:**
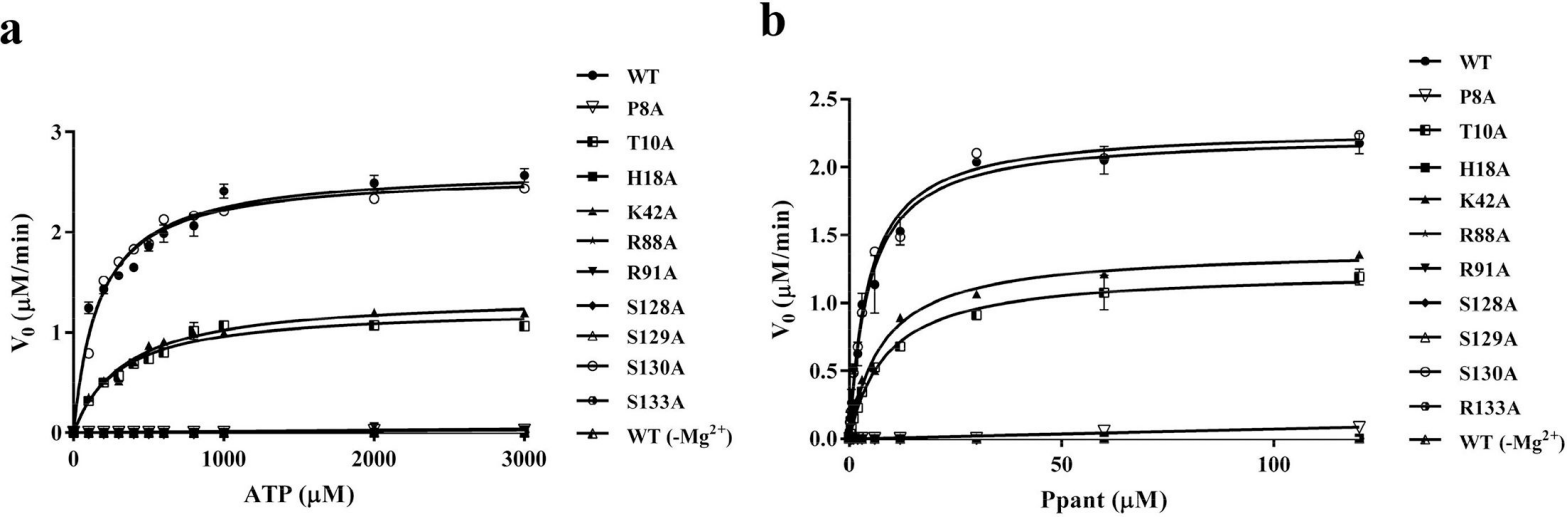
Analysis of comparative steady-state kinetics of the forward reaction of *H. pylori* (*Hp*) WT PPAT and its mutants. The catalytic rate for the *Hp*PPAT forward reaction was measured at various concentrations of ATP and near-saturating Ppant (**a**) or multiple concentrations of Ppant and near-saturating ATP (**b**). Kinetic assays were conducted as detailed in the Materials and Methods section. Each data point represents the mean of three independent replicates, with error bars indicating the standard deviation.

The crystal structure of the *Hp*PPAT:ATP complex does not reveal electron density for a catalytic metal ion (Mg^2+^) associated with the ATP phosphates, similar to the absence of catalytic metal ion density reported in the *Enterococcus faecalis* PPAT complex structure [11]. The enzymatic activity of WT *Hp*PPAT was completely abolished in the absence of Mg²^+^, confirming its essential role in catalysis (Figure 5a and b). This finding supports previous studies on bacterial PPATs, where Mg²^+^ is known to facilitate substrate binding. Forward-reaction assays, detailed in the subsequent section, confirmed that Mg²^+^ is essential for enzymatic activity, indicating the importance of this metal ion despite its absence in the electron density of the crystal structure.

In the *Hp*PPAT:Ppant complex structure, the conserved residues Thr10, Lys42, and Arg88, which are also present in the *Ec*PPAT:Ppant complex structure, form hydrogen bonds with the 4′-phosphate group of pantetheine (Ppant) (Figure 3a, b and g). Amide nitrogen in Thr10 forms a hydrogen bond with non-bridging oxygen in the 4′-phosphate group. In contrast, the hydroxyl group of Thr10 forms a hydrogen bond with another non-bridging oxygen in the same phosphate group. Still, the amino group of Lys42 interacts with the same non-bridging oxygen of Thr10 by forming a hydrogen bond (Figure 3a). The NH_1_ group of Arg88 forms a hydrogen bond with one esterified oxygen of the 4′-phosphate group, whereas the NH_2_ group of Arg88 forms a hydrogen bond with the other esterified oxygen of the 4′-phosphate group. These residues may help orient the nucleophile [12].

To investigate the significance of specific residues in *Hp*PPAT concerning its enzymatic activity, mutants with the following amino acid substitutions were prepared: T10A, H18A, K42A, R88A, R91A, S128A, S129A, and S130A. Additionally, Pro8 in *Hp*PPAT formed a hydrogen bond with the ribose moiety of ATP. In addition, Arg133 established a hydrogen bond with the γ-phosphate group of ATP. These interactions are absent in *Ec*PPAT:ATP and *Ef*PPAT:ATP complexes [11,12]. To investigate the influences of Pro8 and Arg133 on the catalytic step of the forward reaction, mutants P8A and R133A were also prepared. Individual His-tagged mutants were expressed in *E. coli* and purified to homogeneity by cobalt ion affinity chromatography.

To explore the potential influence of the catalytic center region on the *Hp*PPAT structure, we conducted an initial analysis of the kinetic properties of both WT and mutant *Hp*PPAT, focusing on forward reaction activities (Figure 5, Tables 2 and 3). Figure 5 summarizes the results of this investigation, which are detailed in Tables 2 and 3. Our study delved into the steady-state kinetics of the forward reaction using an enzyme-coupled assay. This involved the utilization of inorganic pyrophosphatase to hydrolyze the pyrophosphate generated during PPAT turnover. The resulting orthophosphate was then employed by purine nucleoside phosphorylase (PNP) to catalyze the phosphorolysis of 7-methyl-6-thioguanosine (MESG), resulting in a subsequent increase in absorbance at 360 nm [19,27].

**Table 2:** Comparison of steady-state kinetics of the forward reaction between WT *H. pylori* (*Hp*) PPAT and its mutants using various concentrations of ATP as a substrate

Protein type	Substrate	*k*_cat_ (s^–1^)	*K*_m_ (μM)	*k*_cat_ */K*_m_ (M^-1^ s^-1^)
WT	ATP	1.76 ± 0.09	176.7 ± 36.3	(10.5 ± 2.6) × 10^3^
T10A	ATP	0.83 ± 0.05	301.1 ± 59.9	(2.9 ± 0.7) × 10^3^
K42A	ATP	0.92 ± 0.05	345.9 ± 54.0	(2.6 ± 0.5) × 10^3^
S130A	ATP	1.73 ± 0.07	167.9 ± 30.1	(10.7 ± 2.3) × 10^3^

Note: All values represent the mean ± standard deviation from at least three independent replicates. Other mutants (P8A, H18A, R88A, R91A, S128A, S129A, and R133A) were tested but showed no detectable activity under the same assay conditions (see text).

Parameters determined at a nearly saturating concentration of Ppant.

**Table 3:** Comparison of steady-state kinetics of the forward reaction between WT *H. pylori* (*Hp*) PPAT and its mutants using various concentrations of Ppant as a substrate

Protein type	Substrate	*k*_cat_ (s^–1^)	*K*_m_ (μM)	*k*_cat_ */K*_m_ (M^-1^ s)
WT	Ppant	1.49 ± 0.06	4.6 ± 0.8	(3.4 ± 0.7) × 10^5^
T10A	Ppant	0.82 ± 0.03	8.8 ± 1.2	(0.9 ± 0.2) × 10^5^
K42A	Ppant	0.93 ± 0.05	7.8 ± 1.2	2 ± 0.3) × 10^5^
S130A	Ppant	1.52 ± 0.08	4.5 ± 0.9	(3.6 ± 0.9) × 10^5^

Note: All values represent the mean ± standard deviation from at least three independent replicates. Other mutants (P8A, H18A, R88A, R91A, S128A, S129A, and R133A) were tested but showed no detectable activity under the same assay conditions (see text).

Parameters determined at a nearly saturating concentration of ATP.

Initial reaction rates were calculated under conditions of fixed saturating Ppant concentration (130 μM) while varying ATP concentrations. These data were subsequently fitted to the Michaelis–Menten equation, yielding the following results: K_m (ATP)_= 176.7±36.3 μM and *k*_cat (ATP)_= 1.76±0.09 s^-1^ (Figure 5a). Furthermore, Ppant concentrations were systematically altered while maintaining a constant ATP concentration of 3000 μM. Fitting the initial rates using the same methodology yielded the following values: K_m (Ppant)_ = 4.6±0.8 μM and *k*_cat (Ppant)_ = 1.49±0.06 s^-1^ (Figure 5b). These findings are summarized in Tables 2 and 3. The *k*_cat_/K_m_ values for the forward-direction reaction with ATP and Ppant are similar to those previously reported for *E. coli* PPAT, indicating (7.1±0.2)×10^3^ M^-1^ s^-1^ and (2.9±0.1)×10^5^ M^-1^ s^-1^, respectively [19].

**Figure 6:**
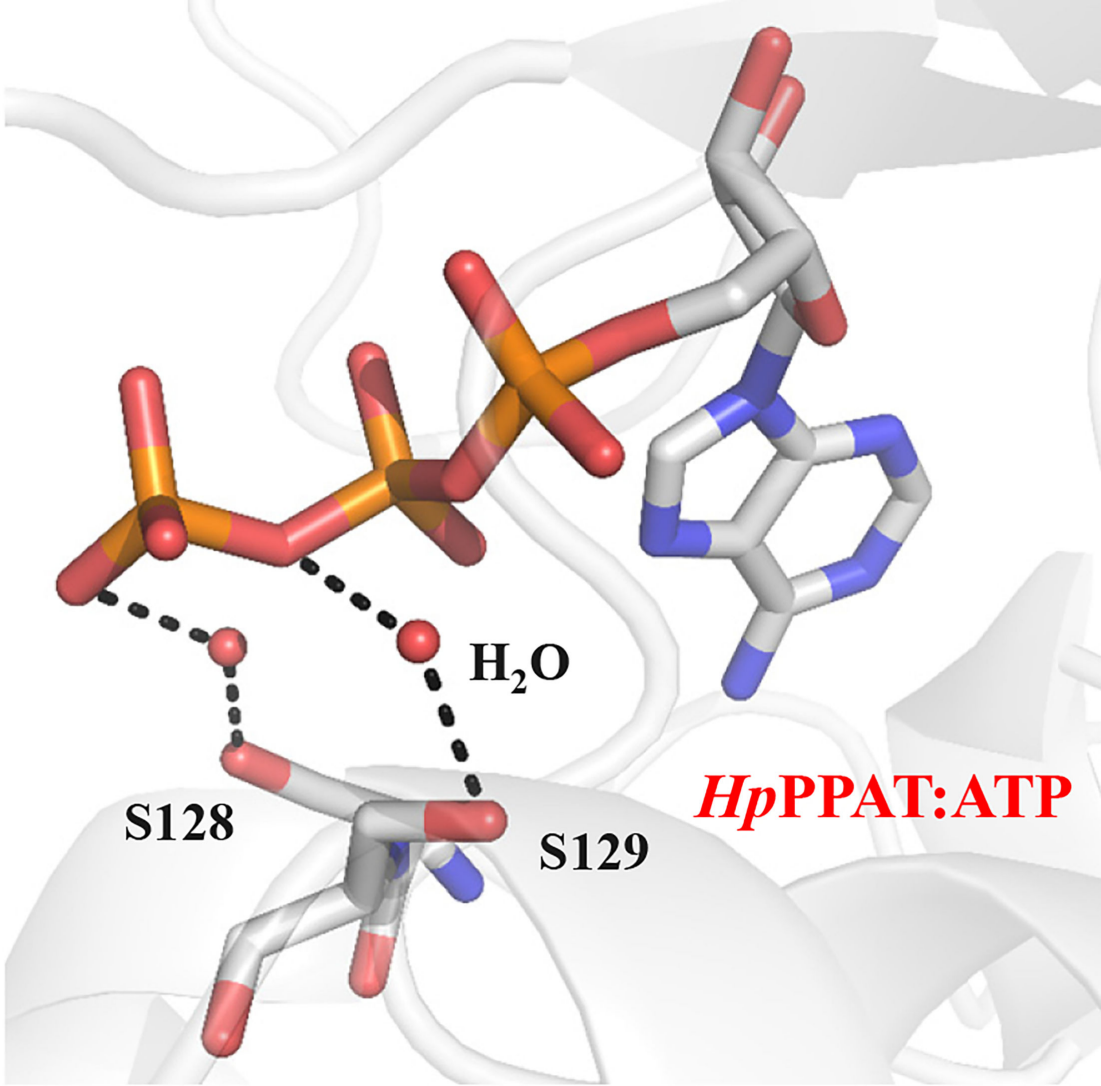
The interactions of Ser128 and Ser129 with ATP in *Hp*PPAT. Ser128 interacts with the γ-phosphate group of ATP (shown as a stick representation) through a water-mediated hydrogen-bonding network. Similarly, Ser129 interacts with the β-phosphate group of ATP via a similar water-mediated hydrogen-bonding network.

The catalytic activities of ten mutants were then assessed in the forward reaction. Mutants P8A, H18A, R88A, R91A, S128A, S129A, and R133A exhibited undetectable responses in Michaelis–Menten kinetics when utilizing ATP and Ppant as substrates, demonstrating a loss of enzyme activity (Figure 5a and b). For *Hp*PPAT, residue Ser128 interacts with the γ-phosphate group of ATP within a water-mediated hydrogen-bonding network, whereas residue Ser129 interacts with the β-phosphate group of ATP in a similar water-mediated hydrogen-bonding network (Figure 6). Disruption of these hydrogen-bonding interactions through site-specific mutagenesis resulted in loss of *Hp*PPAT enzyme activity, underscoring the crucial role of water-mediated interactions in stabilizing phosphates between Ser128, Ser129, and ATP. In the case of the S130A mutant, K_m_ and *k*_cat_ values closely resembled those of the WT concerning ATP and Ppant substrates (Table 2). The structure of the *Hp*PPAT:ATP complex revealed that, unlike *Ec*PPAT Ser130, *Hp*PPAT Ser130 is positioned away from ATP and cannot form a hydrogen bond with the γ-phosphate group. Conversely, *Hp*PPAT Arg133 demonstrates the ability to establish hydrogen bonds with the γ-phosphate group of ATP (Figure 3d). Within *Hp*PPAT, Arg133 may assume the role of stabilizing the γ-phosphate group of ATP instead of Ser130.

T10A and K42A displayed reduced but substantial forward-reaction activities (Figure 5a and b). The apparent *k*_cat_/K_m_ values for substrate ATP in WT, T10A, and K42A mutants were (10.5±2.6) × 10^3^, (2.9±0.7) × 10^3^, and (2.6±0.5) × 10^3^ M^-1^ s^-1^, respectively (Table 2). These values were consistently observed across multiple experiments, confirming that the T10A and K42A mutants exhibit reduced catalytic efficiency compared with the WT *Hp*PPAT, yet their activity remains substantial. The crystal structure of the *Hp*PPAT:Ppant complex revealed that the amide nitrogen of Thr10 forms a hydrogen bond with non-bridging oxygen of the 4′-phosphate group, while the hydroxyl group of Thr10 forms a hydrogen bond with another non-bridging oxygen of the same phosphate group, while the amino group of Lys42 forms a hydrogen bond with the same non-bridging oxygen that interacts with the hydroxyl group of Thr10. The NH1 group of Arg88 forms a hydrogen bond with one of the esterified oxygens of the 4′-phosphate group, while the NH2 group of Arg88 forms a hydrogen bond with the other esterified oxygen of the phosphate group. The partial loss of enzymatic activity upon mutation of Thr10 or Lys42 suggests that these residues contribute essential, but not individually sufficient, stabilizing interactions and nucleophile orientation. These interactions are complementary and cooperative, necessary to maintain significant enzymatic activity and facilitate the in-line displacement mechanism. In contrast, the complete loss of activity observed with the Arg88 mutation highlights its critical role in the process, since Arg88 provides essential stabilization and orientation necessary for the correct positioning and function of the nucleophile in the in-line displacement mechanism. Notably, the hydrogen-bonding interactions between *H*pPPAT Lys42 and ATP occur directly (Figure 3e), whereas in *Ec*PPAT, Lys42 interacts with the α-phosphate group of ATP indirectly through a water-mediated hydrogen-bonding network [12]. This distinction highlights a significant difference in substrate interaction between these enzymes.

#### Biophysical properties of recombinant WT HpPPAT and its mutants

**Figure 7:**
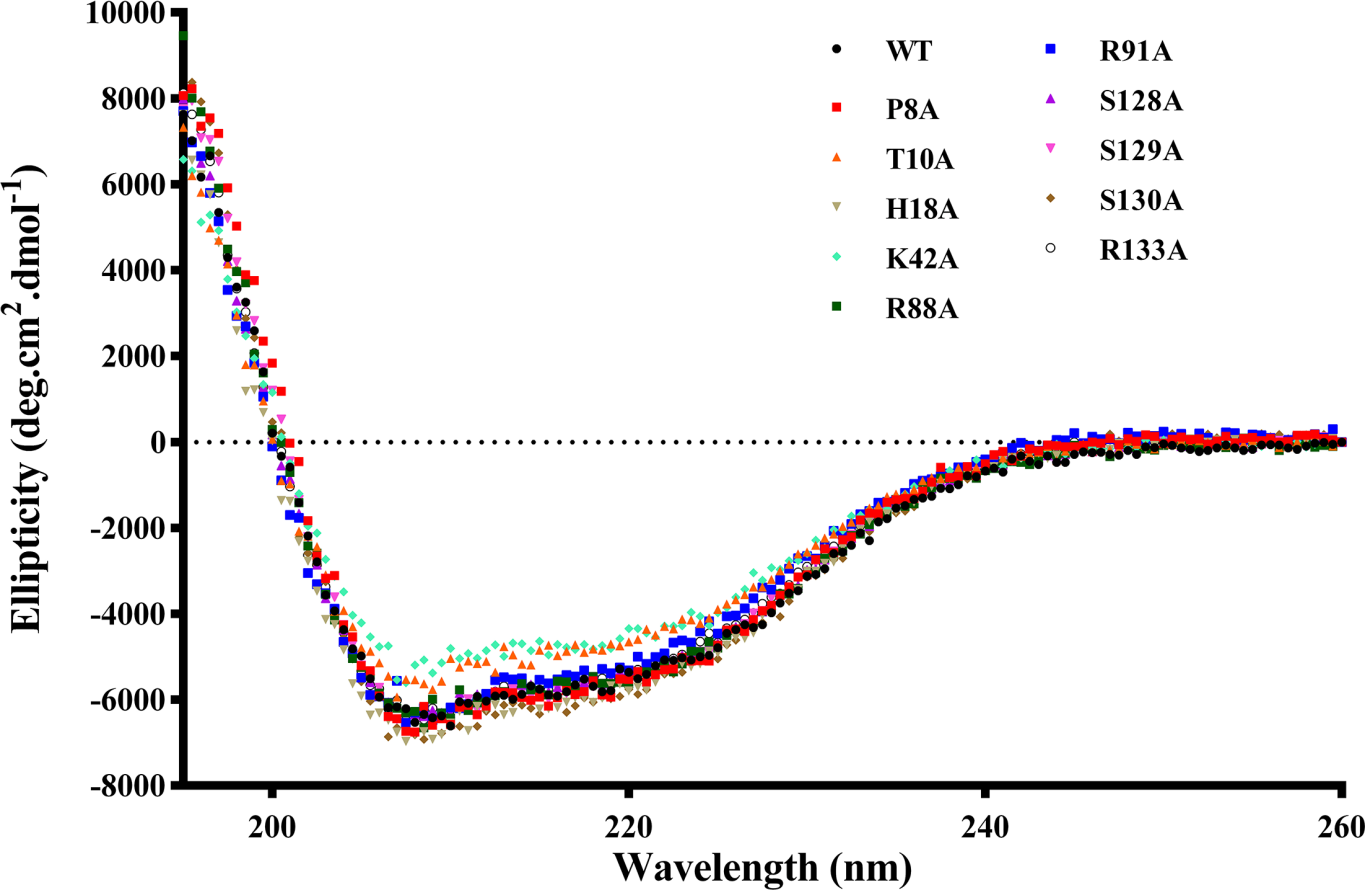
Biophysical properties of recombinant WT *Hp*PPAT and P8A, T10A, H18A, K42A, R88A, R91A, S128A, S129A, S130, and R113A. Secondary structures for recombinant WT and mutant *Hp*PPATs were measured by far-UV CD spectrum.

To determine if the loss of enzymatic activity in mutant proteins is due to misfolding of their secondary structures, circular dichroism (CD) spectroscopy was performed at room temperature. This analysis assessed the secondary structure of WT HpPPAT and its mutants: P8A, T10A, H18A, R88A, R91A, S128A, S129A, and R133A. The spectra for both WT and the mutants displayed a similar pattern, with a maximum at approximately 194 nm and a minimum at approximately 209 nm (Figure 7), consistent with previous studies on *Pyrococcus abyssi* PPAT [28]. These results indicated that the secondary structure of the P8A, T10A, H18A, R88A, R91A, S128A, S129A, and R133A mutants is not misfolded, suggesting that the loss of enzyme functionality is not due to structural misfolding.

#### Structural insights into *Hp*PPAT catalytic mechanism

To elucidate *Hp*PPAT-specific structural insights, we investigated active-site interactions through crystal structures, mutagenesis studies, and kinetic measurements. The catalytic mechanism of *Hp*PPAT follows the well-established reaction pathway described for *E. coli* PPAT, as previously reported [12], rather than proposing a novel mechanism. This reaction mechanism has been characterized in *E. coli* PPAT and involves an in-line nucleophilic displacement process.

Our structural analysis includes three independently determined *Hp*PPAT complex structures. *Hp*PPAT:Ppant and *Hp*PPAT:ATP complexes show the pre-catalytic state of the *Hp*PPAT, illustrating substrate binding before catalysis. In the *Hp*PPAT:Ppant complex (Figure 3a), Thr10, Lys42, Leu74, and Arg88 stabilize the 4′-phosphate of Ppant, positioning it for nucleophilic attack. The *Hp*PPAT:ATP complex (Figure 3d) shows ATP binding stabilized by multiple active-site residues, including Tyr7, Pro8, Thr10, Phe11, His18, Lys42, Arg88, Gly89, Arg91, Asn124, Ser128, Ile127, Ser129, and Arg133. Some of these interactions are species-specific, absent in *E. coli* PPAT (Figure 3e), suggesting functional differences in ATP binding. The *Hp*PPAT P8A mutant:dPCoA complex captures the post-catalytic state, representing the enzyme-product conformation. Structural analysis reveals that residues previously stabilized ATP, including Thr10, Lys42, Leu74, and Arg88, remain interacting with dPCoA (Figure 4b), suggesting their involvement in product stabilization. Similarly, residues that initially stabilized ATP, such as Tyr7, Phe11, Arg88, Gly89, Arg91, Asn124, and Ile127, continue to interact with dPCoA (Figure 4b), indicating their functional role in maintaining the enzyme–product complex before product release.

**Figure 8:**
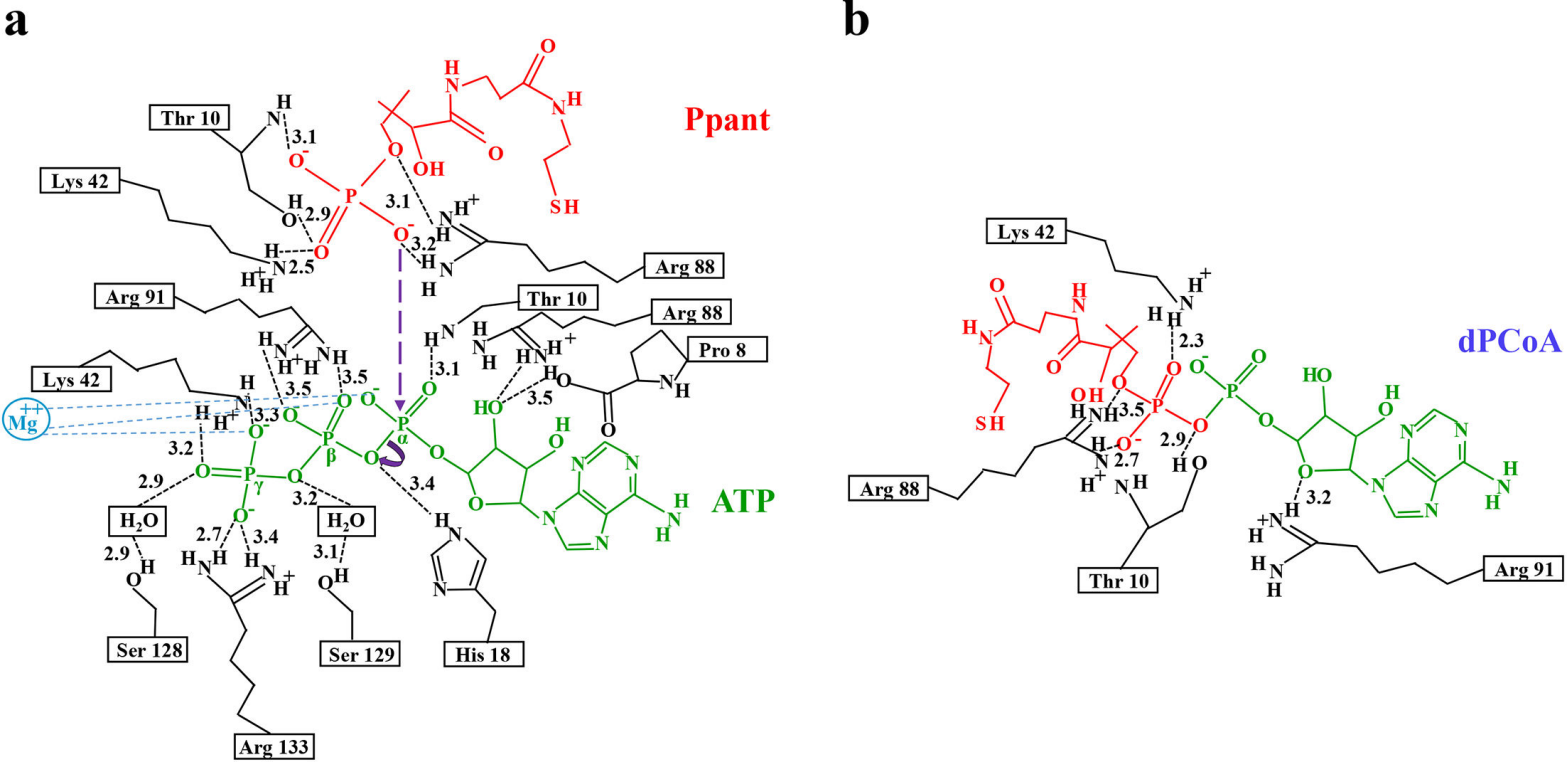
Structural transitions and catalytic co-ordination in *Hp*PPAT. This figure illustrates the pre-catalytic (substrate binding) and post-catalytic (product binding) states of *Hp*PPAT. Ppant (red), ATP (green), and dPCoA (red/green) are derived from separate *Hp*PPAT (black) crystal structures, representing distinct reaction stages. (**a**) The pre-catalytic state is represented by the *Hp*PPAT:Ppant and *Hp*PPAT:ATP complexes, which show substrate binding before catalysis. Lys42, Thr10, and Arg88 stabilize Ppant’s 4′-phosphate, positioning it for phosphotransfer. His18 interacts with ATP’s α-phosphate, and probably provides stability to the later transition state. The purple dashed arrow represents the expected nucleophilic attack pathway, adapted from *E. coli* PPAT [12], rather than a direct structural observation. (**b**) The post-catalytic state is displayed by the *Hp*PPAT P8A mutant:dPCoA complex, which captures the enzyme–product conformation. Active-site residues (Thr10, Lys42, and Arg88) remain engaged with dPCoA, while residues previously stabilizing ATP (Arg88 and Arg91) continue interacting with dPCoA, which may reflect altered substrate interaction patterns during product retention. Mg²^+^ (cyan), absent in our *Hp*PPAT:ATP structure, has been incorporated based on its established role in ATP co-ordination, interacting with ATP’s α-, β-, and γ-phosphate oxygens (cyan dashed lines) and it is proposed to give stability to the later transition state. Hydrogen-bonding interactions are labeled, with interatomic distances (Å) indicated. The multiple representations of Arg88, Lys42, and Thr10 reflect their distinct roles in different catalytic phases rather than suggesting an alternative reaction mechanism. For clarity, only residues experimentally validated through mutagenesis are shown in **Figure 8**. Other residues, including Leu74, Tyr7, Phe11, Gly89, Asn124, and Ile127, also form hydrogen bonds with substrates or products (as shown in Figures 3 and 4), but their functional significance was not tested in this study.

Because the *Hp*PPAT:Ppant and *Hp*PPAT:ATP structures were determined separately, the nucleophilic attack distance between Ppant’s 4′-phosphate oxygen and ATP’s α-phosphate could not be directly measured. The purple dashed arrow in Figure 8 represents the expected nucleophilic attack pathway, modeled based on the *E. coli* PPAT mechanism rather than direct structural observation.

Although the overall catalytic mechanism remains conserved, our study highlights species-specific differences in *Hp*PPAT’s active-site interactions that influence enzymatic activity. Mutagenesis experiments show that alanine substitutions at Thr10, His18, Lys42, Arg88, Arg91, Ser128, Ser129, and Arg133 significantly reduced or abolished enzymatic activity. This confirms their essential roles in catalysis. Structural analysis also reveals that ATP’s β- and γ-phosphate groups are precisely positioned in hydrogen-bonding networks. Arg91, Ser128, Ser129, and Arg133 contribute to this stabilization through specific interactions. Ser129 forms a water-mediated hydrogen bond with ATP’s β- and α-phosphate oxygens, while Ser128 establishes a water-mediated hydrogen bond with ATP’s γ-phosphate oxygens, ensuring a catalytically favorable conformation for substrate binding.

Although Mg²^+^ was not observed in our *Hp*PPAT:ATP complex, it was previously reported in *E. coli* PPATs. It co-ordinates with ATP’s α-, β-, and γ-phosphate groups and is proposed to give stability to the later transition state. This role has been illustrated in Figure 8, where Mg^2+^ interacts with ATP via cyan dashed lines, supporting its function in phosphoryl transfer. To improve clarity, we have revised Figure 8 to clearly distinguish between the pre-catalytic state (substrate binding, Figure 8a) and the post-catalytic state (product binding, Figure 8b), ensuring a more precise representation of *Hp*PPAT’s catalytic process while maintaining alignment with the well-characterized bacterial PPAT mechanism.

## Conclusion

This study elucidates key residues’ structural and functional roles in *Hp*PPAT by presenting crystal structures and analyzing mutants. Our findings offer insights into substrate binding, residue contributions, and *Hp*PPAT’s catalytic mechanism. The side chains of Lys42, Thr10, and particularly Arg88 are crucial for stabilizing the 4′-phosphate group of Ppant and orienting the nucleophile for an in-line displacement mechanism. We also identified the critical roles of Pro8, Lys42, and Arg133 in ATP binding and catalysis. Alanine mutations of Pro8, Lys42, and Arg133 lead to loss of function. The crystal structure of *Hp*PPAT in complex with dPCoA at pH 7.4 shows that dPCoA occupies all hexameric sites of *Hp*PPAT, which suggests that the enzyme does not exhibit half-of-sites reactivity. This research provides valuable insights for developing targeted therapies against *H. pylori* infections.

## Materials and methods

### Materials

ATP, Luria–Bertani broth, kanamycin, imidazole, sodium chloride, and tris(hydroxymethyl)aminomethane (Tris) were supplied by USB (Cleveland, OH). Lithium sulfate, fomblin oil, and 2-mercaptoethanesulfonic acid were purchased from Sigma-Aldrich (St. Louis, MO). Polyethylene glycol 1000, sodium acetate, and citrate phosphate were sourced from Hampton Research (Aliso Viejo, CA). The strain of *E. coli* BL21 (DE3) was obtained from Yeastern Biotech (cat. #YE207) in Taipei, Taiwan. Isopropyl β-D-1-thiogalactopyranoside was purchased from Protech (Taipei, Taiwan). 4′-phosphopantothenate (Ppant) was supplied by Enamine Ltd. (Kyiv, Ukraine).

### Gene cloning and mutagenesis

The gene encoding WT *Hp*PPAT was cloned into vector pET-28(+) (cat. #69864) from Novagen (Whitehouse Station, NJ), as outlined in a previous study [29]. Site-directed mutagenesis was performed using the Quikchange kit (cat. #200516) from Stratagene (The Netherlands) to replace the codons for residues P8, T10, T15, H18, K42, K88, R91, Y98, S128, S129, S130, and R133 with alanine. The mutated *Hp*PPAT genes were individually cloned into pET-28a(+) with an upstream T7 promoter-His6 tag and expressed in *E. coli* BL21 (DE3). DNA sequencing confirmed gene sequences (Mission Biotechnology Inc., Taiwan). Supplementary Table S1 provides details about the primer.

### Bacterial culture and protein purification

Cells were cultured in Luria–Bertani medium containing 50 μg/ml kanamycin. Induction of protein expression occurred at an OD_600_ of 0.6 with 4 mM isopropyl-thio-β-D-galactopyranoside. Protein purification and molecular masses were characterized by SDS-PAGE [30] and the Autoflex III MALDI-TOF mass spectrometer (Bruker Daltonics Inc., Billerica, MA) [31]. Protein concentrations were measured using the Quick Start Bradford Protein Assay kit (cat. #5000201) from Bio-Rad (CA), with bovine serum albumin as the standard. *Hp*PPATs (15 mg/ml) in 25 mM Tris-HCl, 100 mM NaCl, pH 7.4 served as stock solutions.

### Crystallization, data collection, and refinement

The apo *Hp*PPAT, *Hp*PPAT:ATP, *Hp*PPAT:Ppant, and *Hp*PPAT mutant P8A (P8A):dPCoA crystals were formed in greased wells of 48-well plates (Hampton Research, Aliso Viejo, CA) using the hanging-drop vapor-diffusion crystallization method and protein (15 mg/ml) in a protein solution (20 mM Tris-HCl, pH 7.9, 100 mM NaCl) at 293K. The apo *Hp*PPAT crystals were grown in a reservoir solution (2.5 M NaCl, 0.1 M sodium acetate pH 4.5, and 0.2 M Li_2_SO_4_) within two weeks. Ppant-bound *Hp*PPAT crystals were grown by soaking apo crystals in a reservoir containing 3 mM Ppant for 2 h. Crystals of *Hp*PPAT in complex with ATP were obtained in a reservoir solution of 17% PEG1000, 0.1 M citrate phosphate pH 4.2, 1.5 mM ATP, and 0.2 M Li_2_SO_4_ within three days. Crystals of *Hp*PPAT mutant P8A in complex with dPCoA were grown in a reservoir solution of 18% PEG550 MME, 0.2 M KF pH 7.4 within one week. All crystals were transferred to Fomblin oil as cryoprotectants and frozen in liquid nitrogen for data collection. X-ray diffraction data were collected at the SPXF beamline BL13B1 of the National Synchrotron Radiation Research Center in Hsinchu, Taiwan, using a mar345 Image Plate Detector (Marresearch GmbH, Germany). Structure calculation, refinement, and validation followed established protocols [32]. Diffraction datasets were processed using the HKL-2000 package (HKL Research Inc., Charlottesville, VA) [33]. The crystals for apo *Hp*PPAT and *Hp*PPAT:Ppant belong to space group *H*3, and the asymmetric unit for both contains two copies of *Hp*PPAT. The crystals for *Hp*PPAT:ATP belong to space group *P*1, and the asymmetric unit has 12 copies of *Hp*PPAT. P8A:dPCoA possesses crystallographic two- and three-fold symmetry and is a hexamer belonging to space group *P*2_1_2_1_2. Unit-cell dimensions, data collection, and crystallographic parameters are summarized in Table 1.

Molecular replacement was performed to generalize the model of apo *Hp*PPAT with the CCP4 program Molrep [34] with the *Hp*PPAT:CoA complex structure [Protein Data Bank (PDB) ID 3OTW] [18] as the search model. Data between 25.0 and 3.0 Å and a Patterson radius of 20 Å were used to calculate the rotation and translation functions. In the same way, apo *Hp*PPAT was used as a template to build the structures for *Hp*PPAT:Ppant, *Hp*PPAT:ATP, and P8A:dPCoA. The initial apo *Hp*PPAT model was refined with Refmac5 [35] and rebuilt with Coot [36]. Structure validation was performed using PROCHECK v.3.5.4 [37], and secondary structures were identified by DSSP [38]. Table 1 summarizes the crystallographic data and refinement statistics. The atomic co-ordinates and structure factors have been deposited in the PDB under accession codes 8XSK (apo *Hp*PPAT)[39], 8XRW (*Hp*PPAT:ATP[40]), 8XSC (*Hp*PPAT:Ppant)[41], and 8XSV (*Hp*PPAT mutant P8A)[42]. Molecular graphics software PyMOL (DeLano Scientific; http://www.pymol.org) and Discovery Studio 4.0 (Accelrys Inc., San Diego, CA) were employed for molecular visualization.

## Enzyme assays

The catalytic activity of WT *Hp*PPAT and mutants (25 nM) was measured by using EnzChek Pyrophosphate Assay kit (cat. #6645) from Thermo Fisher Scientific (Waltham, MA), based on a method described initially by Webb that monitors the amount of free phosphate present [13,27]. Each forward reaction contained 20 mM Tris-HCl (pH 7.9), 125 mM NaCl, 6 mM MgCl_2_, PNP (1 U/ml), inorganic pyrophosphatase (0.03 U/ml), 2-amino-6-mercapto-7-methylpurine ribonucleoside (MESG) (0.2 mM), and tris(2-carboxyethyl) phosphine hydrochloride (1 mM). Pyrophosphate formed by PPAT turnover is cleaved into two orthophosphate molecules by inorganic pyrophosphatase. This is then consumed by PNP, leading to the phosphorolysis of MESG and an increase in absorbance at 360 nm. Various concentrations of Ppant (ranging from 0 to 120  μM) and ATP (ranging from 0 to 3000  μM) were added to assess substrate-dependent kinetics. Magnesium was maintained at a minimum of 6  mM, or at least twice the highest ATP concentration used [19]. The absorbance of each reaction was measured in a 1-cm path-length cuvette at 360 nm for 120 s at 25°C, using a Hitachi U-3300 UV–VIS spectrophotometer (Tokyo, Japan).

## Kinetic analysis

UV-visible data were converted to initial velocity using Excel 2016 (Microsoft), and kinetic parameters were determined by fitting the data to the Michaelis–Menten equation using nonlinear regression and Prism software version 7.2 for Windows (GraphPad Software, La Jolla, CA). Assays were repeated at least three times, with upper and lower error bars representing duplicate measurements and each data point representing the mean.

### Circular dichroism spectroscopy

CD spectra were analyzed to estimate protein secondary structure and stability using an Aviv 202 spectropolarimeter (Aviv Biomedical Inc., Lakewood, NJ, U.S.A.) [43]. WT *Hp*PPAT or mutants P8A, T10A, T15A, H18A, R88A, R91A, S128A, S129A, and R133A proteins (15 μM) in 10 mM potassium phosphate buffer (pH 7.4) were measured at 25°C in the far-UV region (195–260 nm) using a 1-mm path-length cuvette. Three CD scans were averaged, and spectra are reported as mean residue ellipticity (θ) in deg cm^2^ dmol^-1^.

## Supplementary Material

Online supplementary figures

## Data Availability

The authors confirm that the data supporting this study’s findings are available in the article and/or its supplementary materials. The atomic co-ordinates and structure factors have been deposited in the Protein Data Bank with accession codes 8XSK (apo HpPPAT), 8XRW (HpPPAT:ATP), 8XSC (HpPPAT:Ppant), and 8XSV (HpPPAT mutant P8A).

## Abbreviations

CD: circular dichroism
CoA: coenzyme A
EcPPAT: E. coli PPAT
HpPPAT: H. pylori PPAT
HpPPAT: Helicobacter pylori PPAT
MESG: 7-methyl-6-thioguanosine
MtPPAT: Mycobacterium tuberculosis PPAT
PNP: purine nucleoside phosphorylase
PPAT: Phosphopantetheine adenylyltransferase
Ppant: phosphopantetheine
dPCoA: dephospho-CoA
