# Supplementary material for: Structural insights into substrate binding, residue contributions, and catalytic mechanism of phosphopantetheine adenylyltransferase from Helicobacter pylori
Source: Biosci Rep. 2025 Aug 26;45(8):19–34. doi: 10.1042/BSR20241405 (PMC12784347; doi:10.1042/BSR20241405)

# Supplementary File

Structural insights into substrate binding, residue contributions, and catalytic mechanism of phosphopantetheine adenylyltransferase from *Helicobacter pylori*

I-Ting Ko^†^, Yi-Ting Yuan^†^, Cheng-Ju Hsieh, and Hsien-Sheng Yin*

Institute of Bioinformatics and Structural Biology, and College of Life Sciences, National Tsing Hua University, No. 101, Section 2, Kuang-Fu Road, Hsinchu 30013, Taiwan

*Corresponding author: Hsien-Sheng Yin ([hstin@mx.nthu.edu.tw](mailto:hstin@mx.nthu.edu.tw).)
†: Equal contribution

Institute of Bioinformatics and Structural Biology, and College of Life Sciences, National Tsing Hua University, No. 101, Section 2, Kuang-Fu Road, Hsinchu 30013, Taiwan

E-mail: hstin@mx.nthu.edu.tw; Tel: +886-3-574-2469; Fax: +886-3-571-5934

Keywords: phosphopantetheine adenylyltransferase, *Helicobacter pylori*, enzyme kinetics, crystal structure

**Supplementary Table 1.** Primers used in this study. The primer sequences used for site-directed mutagenesis of *Hp*PPAT mutants are listed. Each entry includes forward and reverse primers designed to generate the specified amino acid substitutions.

| PPAT gene | Forward Primer (5' → 3') | Reverse Primer (5' → 3') |
| --- | --- | --- |
| P8A | aaaatcggcatttacgccggcactttt | cggatcaaaagtgcccgggataatgcc |
| T10A | ggcatttacccgggcgcctttgatccg | agtcagcggatgaaacgggcccgggta |
| H18A | ccggtcactaacggggccatagacatt | aatgtctatggccccgttagtgaccgg |
| K42A | gcacattcaagcgctgccaaccctatg | actaaacatagggttggcagcgcttga |
| R88A | tgtaaggtgttagttgccggtttaagg | CACCACCCTTAAACCGGCAACTAACAC |
| R91A | TTAGTTAGGGGTTTAGCCGTGGTGAGC | aaaatcgctcaccacggctaaacccct |
| S128A | CAAAACGCTTTCATAGCCTCTTCTATC | ACGCACGATAGAAGAGGCTATGAAAGC |
| S129A | AACGCTTTCATAAGCGCCTCTATCGTG | GGATCGCACGATAGAGGCGCTTATGAA |
| S130A | GCTTTCATAAGCTCTGCCATCGTGCGA | AATGGATCGCACGATGGCAGAGCTTAT |
| R133A | AGCTCTTCTATCGTGGCCTCCATTATC | ATGCGCGATAATGGAGGCCACGATAGA |

**Supplementary Figure 1.** Electron density maps of ATP in the *Hp*PPAT:ATP complex. This figure illustrates the electron density of ATP in fully and partially occupied monomers of the *Hp*PPAT:ATP complex at 2.12 Å resolution. (a) ATP exhibits well-defined electron density in the fully occupied monomer (chain L). (b) In contrast, the partially occupied monomer (chain K) shows fragmented ATP density, indicating partial occupancy. The 2*Fo-Fc* electron density maps are contoured at 1.0σ, with ATP displayed in stick representation.


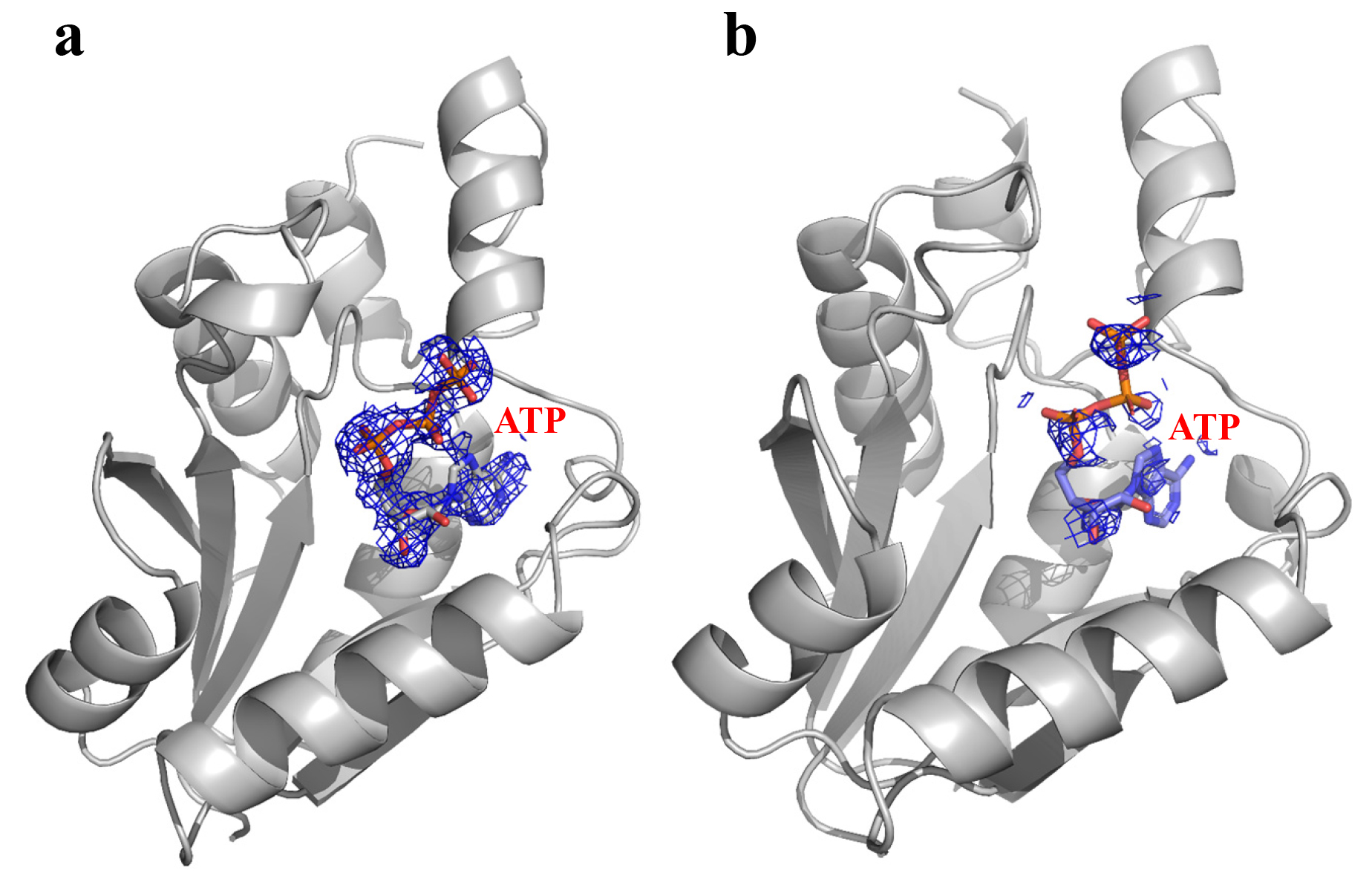


**Supplementary Figure 2.** Comparative ATP production in the reverse reaction by WT *Hp*PPAT and the P8A mutant. The reverse catalytic activity of wild-type (WT) *Hp*PPAT and the P8A mutant was assessed using an ATP determination kit (A22066I, Invitrogen), which utilizes firefly luciferase to quantify ATP formation. In this reaction, *Hp*PPAT catalyzes the adenylation of dephospho-coenzyme A (dPCoA) by pyrophosphate (PPi), producing ATP and phosphopantetheine (Ppant). The luminescence intensity, measured using a multi-label microplate reader (Perkin Elmer Victor3, USA), correlates with ATP production, enabling comparative evaluation between WT and mutant. Michaelis-Menten kinetics was applied under saturating concentrations of dPCoA and PPi, with varying enzyme concentrations. ATP production was quantified and normalized to WT activity. As shown, the P8A mutant exhibited significantly higher ATP production than WT *Hp*PPAT, suggesting enhanced catalytic efficiency. PBS was used as a negative control. Error bars represent the standard deviation (SD) from at least three independent experiments. Statistical analysis using an unpaired t-test (*p* < 0.01) confirmed the significant increase in ATP production by the P8A mutant.

**
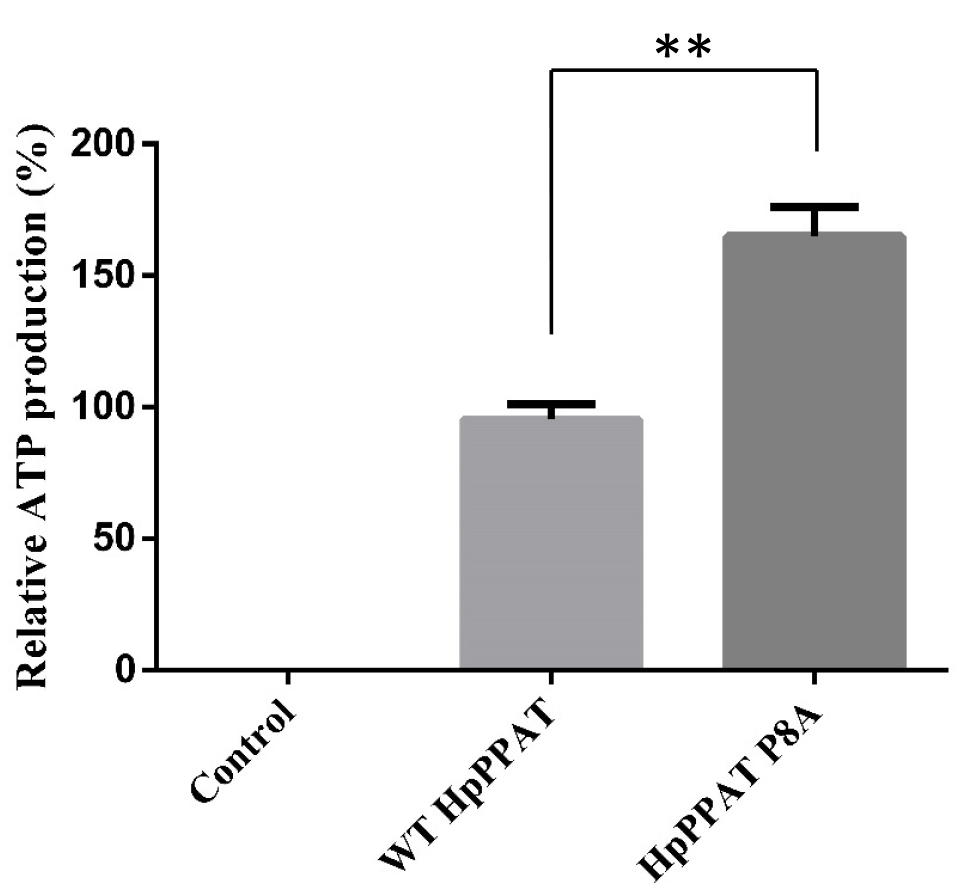
**

**Supplementary Figure 3.** SDS-PAGE gel (15% (w/v) acrylamide) stained with Coomassie blue of purified recombinant WT *Hp*PPAT and its mutants. All lanes were loaded with 10 μg of protein. The molecular masses of the protein standards (kDa, lane M) are shown to the left of the gel. Lane: 1, WT *Hp*PPAT; 2, P8A; 3, T10A; 4, H18A; 5, K42A; 6, R88A; 7, R91A; 8, S128A; 9, S129A; 10, S130A; 11, R133A.


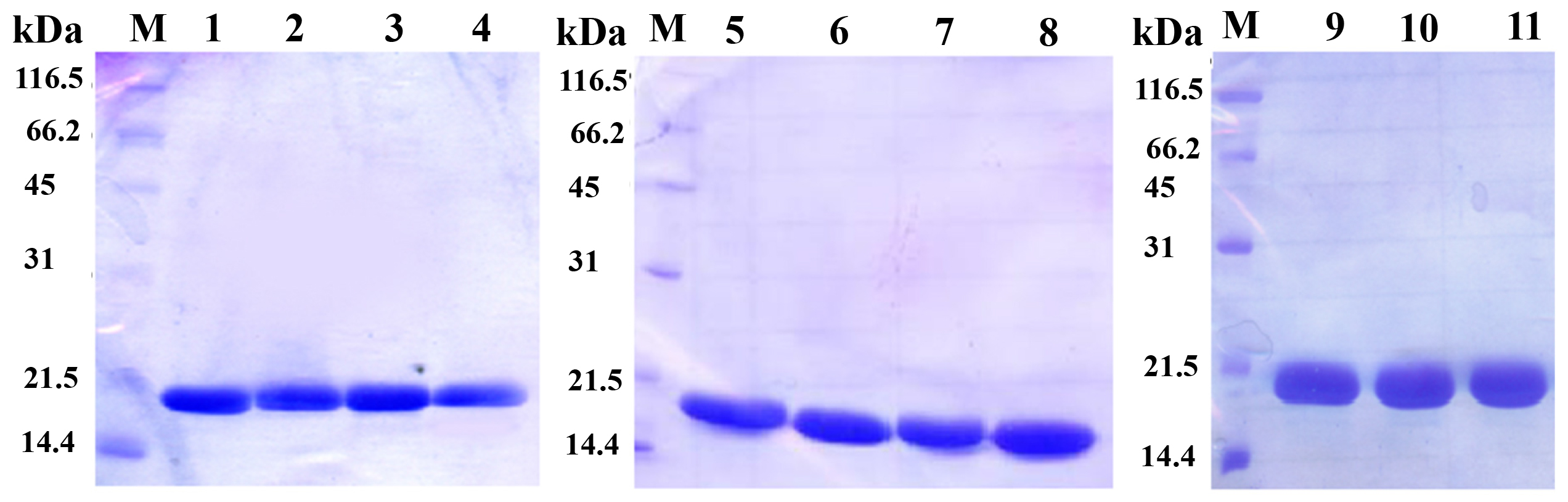

Supplement: Online supplementary figures [file bsr-45-08-BSR20241405-s001.doc]
